# Immunopathological Changes in SARS-CoV-2 Critical and Non-critical Pneumonia Patients: A Systematic Review to Determine the Cause of Co-infection

**DOI:** 10.3389/fpubh.2020.544993

**Published:** 2021-02-09

**Authors:** Saikat Samadder

**Affiliations:** The Oxford College of Science, Bengaluru, India

**Keywords:** SARS-CoV-2, COVID-19, cytokines, lymphocyte, neutrophil, viral pneumonia, interleukins, interferon

## Abstract

The ongoing COVID-19 pandemic originating from Wuhan, China is causing major fatalities across the world. Viral pneumonia is commonly observed in COVID-19 pandemic. The number of deaths caused by viral pneumonia is mainly due to secondary bacterial or fungal infection. The immunopathology of SARS-CoV-2 viral pneumonia is poorly understood with reference to human clinical data collected from patients infected by virus and secondary bacterial or fungal infection occurring simultaneously. The co-infection inside the lungs caused by pneumonia has direct impact on the changing lymphocyte and neutrophil counts. Understanding the attribution of these two immunological cells triggered by cytokines level change is of great importance to identify the progression of pneumonia from non-severe to severe state in hospitalized patients. This review elaborates the cytokines imbalance observed in SARS-CoV-1 (2003 epidemic), SARS-CoV-2 (2019 pandemic) viral pneumonia and community acquired pneumonia (CAP), respectively, in patients to determine the potential reason of co-infection. In this review the epidemiology, virology, clinical symptoms, and immunopathology of SARS-CoV-2 pneumonia are narrated. The immune activation during SARS-CoV-1 pneumonia, bacterial, and fungal pneumonia is discussed. Here it is further analyzed with the available literatures to predict the potential internal medicines, prognosis and monitoring suggesting better treatment strategy for SARS-CoV-2 pneumonia patients.

## Introduction

The pandemic of severe acute respiratory syndrome coronavirus 2 (SARS-CoV-2) is currently causing a huge number of deaths globally, identified on 1st December 2019 in Wuhan, China (1). The SARS-CoV-2 emerges from Riboviria realm belonging to the coronaviridae family under the genus betacoronavirus responsible for global deaths causing SARI (severe acute respiratory infection) in humans (2). The SARS-CoV-2 infection can rapidly infect immunosuppressed patients and aging individuals were considered to be at higher risk of acquiring coronavirus disease-19 (COVID-19) (3). The entry of this virus is responsible for causing secondary bacterial or fungal co-infection increasing the criticality in patients (4). On 11th February 2020 World Health Organization (WHO) named the disease caused by coronavirus as COVID-19 and changed the virus name from 2019-nCoV to SARS-CoV-2 due to its high similarity with epidemic SARS (SARS-CoV-1) coronavirus (5). Presently COVID-19 has spread across all the continents except Antarctica (6).

The transmission of coronavirus across the species is possible through antigenic shift (7). Where possibly two viral antigenic genes recombined within the host cell gives rise to a new epidemic virus, previously observed during influenza virus pandemic (8). A study conducted in India in collaboration with China (Wuhan institute of virology), USA and Singapore (published in August 2019) suggested that bats are natural reservoir for several viral species including Ebola and Marburg virus (9). Coronavirus similar to influenza virus causes upper and lower respiratory tract infection (LRTI), it gradually in association with bacterial superinfection results in severe lower respiratory tract infection giving rise to fatal condition (10). SARS-CoV-2 is responsible for upper and LRTI causing severe acute respiratory infection (SARI) (3). Similar to SARS-CoV-1, it leads to acute lung injury (ALI) and acute respiratory distress syndrome (ARDS) further progresses to sepsis or myocardial injury resulting in death of the patient (11, 12). Apart from respiratory failure and sepsis as major cause of raising death toll, myocarditis, acute myocardial injury, ischemic stroke related deaths and multiple organ failure like acute kidney injury are being seen in COVID-19 patients (13–15).

## Scope and Focus

The immune activation during pneumonia caused by fungi or bacteria or co-infection seen in viral pneumonia patients is highlighted here. The main purpose of this review article is to identify the possible cause of secondary pneumonia infection observed in COVID-19 patients. In this review the overall immunopathological changes and immune imbalance observed previously during SARS-CoV-1 pneumonia epidemic, presently in SARS-CoV-2 pneumonia pandemic, moderate and severe infection, respectively, are discussed in detailed manner to reveal the potential cause of superinfection. Beside that immune activation during community acquired pneumonia (CAP) caused by fungi or bacteria or co-infection seen in SARS-CoV-2 virally challenged patients are highlighted. Although there is no major difference in terms of cytokine storm observed in SARS-CoV-1 and SARS-CoV-2 patients however to understand if dysbiosis causes superinfection is reviewed. This review would be helpful in distinguishing the role of anti-inflammatory drugs during the progression of moderate to severe form of SARS-CoV-2 pneumonia caused by cytokine storm.

At present there are several scientific questions arising from the existing pandemic situation. The reason behind children and women are less fatally challenged by COVID-19 than the aged males. The potential reason why majority of patients survive COVID-19 disease without any complications while others struggle or die irrespective of known comorbidities is reviewed here. This article would further help to design a better clinical investigational plan for studying and monitoring viral pneumonia in patients. Here the significance of reporting the basic cytokines level in serum or bronchoalveolar lavage (BAL) fluids of SARS-CoV-2 pneumonia patients is briefly discussed. Apart from that epidemiology, virology, clinical symptoms, prognosis, therapeutic interventions and potential limitations are discussed in this detail review article along with the immunopathology and cytokine imbalance observed in SARS-CoV-2 severe and non-severe pneumonia patients.

## Methodology

**Figure 1:**
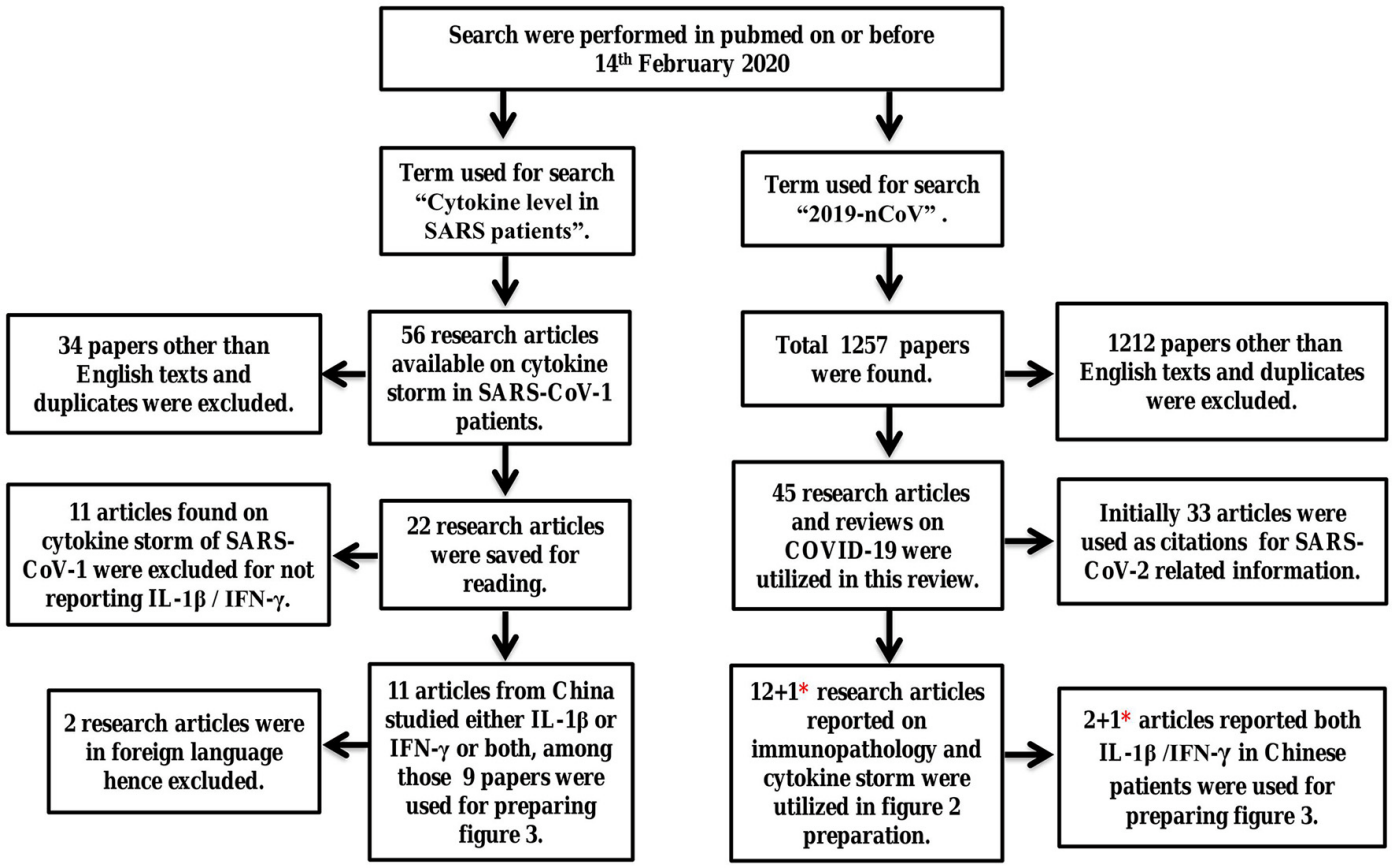
Systematic review of literature to study the immunopathology and immune imbalance resulting in pneumonia. Here the term SARS refers to SARS-CoV-1 and 2019-nCoV refers to SARS-CoV-2 virus. *One research article on SARS-CoV-2 published in April 2020 was added later to both Tables 1, 2.

To find the potential cause of pneumonia in SARS-CoV-2 infected patients, systematic review of literature was performed and the procedure of Ahmed et al. was followed (16). Using the terms “cytokine level in SARS patients” and “2019-nCoV” pubmed was searched on or before 14th February 2020, results obtained as 56 and 1,257 published articles respectively. All the research articles reported on SARS-CoV-1 and SARS-CoV-2 patients published between 1st January 2003 till 14th February 2020 and 1st December 2019 till 14th February 2020, respectively, were reviewed. Duplicate articles and literatures other than English language were excluded. The articles reported on virology immunopathological changes, symptoms and cytokine profiles in COVID-19 patients were included. After following the inclusion and exclusion criteria 22 studies on SARS-CoV-1 and 45 research articles on SARS-CoV-2 were selected and saved for reading. The studies emphasizing on cytokines levels of either interleukin-1beta (IL-1β) or interferon-gamma (IFN-γ) or both in SARS-CoV-1 and SARS-CoV-2 infected Chinese patients was the inclusion criteria and selected in **Table 2**. The reason for choosing this criterion is discussed in paragraph 12 & 15. Upon implementation of the inclusion criteria, 9 papers on SARS-CoV-1 were selected for preparation of **Table 2**. Similarly, 12 research articles on immunopathological changes and cytokine profiles of COVID-19 patients were presented in Table 1. Among those two articles were utilized in Table 2 to describe cytokine imbalance of IL-1β/IFN-γ. One research article related to cytokine storm in COVID-19 patients published in April 2020 was added in Tables 1, 2 as its findings demonstrated cytokine imbalance of IL-1β and IFN-γ (23). To ensure that updated information on CAP, SARS-CoV-1, and COVID-19 are reviewed here google authors, pubmed, WHO, and center for disease control and prevention (CDC), respectively, were searched with appropriate terms or questions during manuscript preparation. Methodology is described with a flow chart in Figure 1.

**Table 1:** Represents number of patients included in each studies immunopathological changes reported in COVID-19 (2019-nCoV) infected patients.

**Study**	**Number of patients**	**Immunopathological changes (Lymphocytes and neutophils counts)**	**Cytokine and chemokine levels in COVID-19 patients**	**References**
1	1	Mild changes in blood like leukopenia and thrombocytopenia.	NA	(17)
2	2	Lymphocyte, Neutophil, WBC counts were in normal range for patient 1, in Patient 2 lymphocyte count reduced.	NA	(18)
3	4	Neutophil count reduced in discharged adult patients upon treatment but patients severely affected had high neutrophil and low lymphocyte count.	NA	(19)
4	6	Adult patient displayed high neutophil level and an elderly patient depleted in neutophils count. Lymphocyte count decreased in two other elderly patients.	NA	(20)
5	9	Average lymphocyte count increased and neutrophil count remained within range in children.	IL-6, IL-17F, IL-22 levels were higher than normal range.	(21)
6	13	Reduced lymphocyte count and neutrophil count in medium range.	NA	(22)
7	41	Highly Significant difference noted in neutophil count in ICU patient compared to Non-ICU, significantly low lymphocyte count in ICU patients.	IL2, IL7, IL10, GSCF, IP10, MCP1, MIP-1α, and TNF-α were significantly high in ICU patients.	(1)
8	50	Significant decrease in lymphocyte count and increase in neutrophil count observed in critically ill patients compared to moderate group.	IP-10, MCP-3, HGF, MIG and MIP-1α levels were high in critical patients (ICU).	(23)
9	99	In 35% patients lymphocyte count decreased and in 38% patients neutrophil count increased during SARS-CoV-2 pneumonia.	IL-6 level was elevated in 52% patients.	(24)
10	137	Lymphocyte count below 1 × 10^9^/L was found in 27% patients.	NA	(25)
11	138	Neutophil count significantly increased in fatal cases than non-fatal cases. Lymphocyte count reduced among fatal cases than non-fatal patients.	NA	(12)
12	191	Lymphocyte count was significantly lower in non-survivor than survivors.	IL-6 level was elevated substantially in non-survivors.	(26)
13	710	No significant changes observed in survivor or non-survivor groups.	NA	(27)

*NA, not available; IL, Interleukins; TNF-α, Tumor necrosis factor-α; IFN**-**γ, Interferon**-**γ; CXCL10/IP-10, IFN**-**γ induced protein 10; MCP-3, monocyte chemotactic protein-3; HGF, hepatocyte growth factor; MIG/CXCL9, monokine induced IFN gamma; MIP-1α, macrophage inflammatory protein 1 alpha*.

**Table 2:** The imbalance of IL-1β and INF-γ/IP-10 in CAP (severe), SARS-CoV-1 (severe and non-severe), and SARS-CoV-2 (severe and non-severe) are shown in this table.

**Diseases and Severity**	**CAP (Severe)**	**SARS-CoV-1 (non-severe)**	**SARS-CoV-1 (severe)**	**SARS-CoV-2 (severe)**	**SARS-CoV-2 (non-severe)**
**CYTOKINES/CHEMOKINE**					
IL-1β	• IL-1β level high in BALF than in serum of fatal cases (28). • Low IL-1β level high in Seven (35%) out of 20 total CAP patients (29). • IL-1β level increased notably in fatal case (30) • IL-1β increased in severe CAP group (31).	• IL-1β level Significantly high in all the patients except three patients during hospitalization compared to normal (32). • IL-1β level was significantly high for 13 days monitored for 20 days of hospitalization (33). • IL-1β did not increase in recovering patients compared to normal (34).	• IL-1β level was (25–49%) higher in fatal cases than normal in lungs demonstrated using IHC (35). • IL-1β level no change in infected patients but increased in a fatal case detected by mRNA expression (31). • 50% increased IL-1β level compared with normal patients (36).	• The IL-1β levels in ICU patients were significant higher than normal but no major difference found in ICU and non-ICU groups (1). • IL-1β level in critically ill patients were significantly low compared with severely ill patients (23).	• Highest statistical significance was observed in IL-1β level of non-ICU patients compared with normal (1). • IL-1β level was in normal range for pediatric patients (21).
IFN-γ/IP-10	• IFN-γ level was significantly higher in Severe CAP patients than non-severe CAP and healthy (29). • IFN-γ level increased in two patients during acute phase but decreased with time (30). • IFN-γ level increased, IP-10 level did not change in pneumonia group (31). • Significantly higher than the SARS-CoV-1 patients 42% patient had CAP (37). • No major difference found in IFN-γ level of CAP and normal patients (38).	• IFN-γ levels not measured (32). • Significantly high for 3 days monitored for 18 days, IP-10 was not monitored (33). • IFN-γ level did not vary in recovering patients compared to normal (34).	• IFN-γ levels in severe and non-severegroups did not vary significantly compared to control group. IP-10 level was high in SARS-CoV-1 patients (31). • IFN-γ level increased by 50% compared to normal (36). • The level of IFN**-**γ was lower than CAP but the IP-10 level was significantly higher than CAP group (37). • Significantly high in fatal than non fatal patients (39) • No Significant change in serum INF-γ level of severe and non-severepatients (40).	• IFN-γ was significantly higher in the ICU patients compared with the healthy individuals (1). • IP-10 level varies with time monitored in critical, severe and moderate pneumonia. Major statistical significance was observed in the critically ill patients and in moderate pneumonia patients after 15 days of infection (23).	• No difference observed in IFN-γ levels of ICU and non-ICU patients but IP-10 was high in ICU but not in non-ICU patients (1). • IFN-γ level was in normal range except for two pediatric patients (21).

*The non-severe group contains hospitalized patients, the severe group patients required frequent oxygen therapy and critical group patients were admitted in ICU under invasive mechanical ventilator*.

## Epidemiology

Particularly while discussing about viral pneumonia neither the death rate of pneumonia nor the viral infections resulting in LRTI could be underestimated. As per WHO, the overall deaths resulted due to LRTI was reported to be three million, excluding tuberculosis related deaths (41). As of 1st Dec 2020 1 year after its outbreak COVID-19 has infected more than 61.8 million individuals which caused above 1.4 million deaths globally while these numbers are still rising steadily. SARS-CoV-2 has spread to several territories more than 200 nations have already reported several cases of COVID-19. As per prediction of WHO 2–5% crude mortality rate would prevail for COVID-19 in world population, at present approximately it is 2.2% after 1 year (6). The ongoing pandemic situation raised by COVID-19 will put additional burden on overall global death rate caused by infectious diseases. Unlike Influenza or SARS-CoV-1, the current pandemic COVID-19 is causing lesser number of deaths in children (26). Men are at higher risk than female exposed to SARS-CoV-2 the exact reason is unknown. At the same time smoking was not found to be a predisposing factor for the patient's complications (42). Due to presence of co-morbidities like age, gender, diabetes, anemia and immunosuppressed or immunocompromised conditions including HIV or cancer, respectively, increases the chance of fatality (24).

As of 1st December 2020, countries like USA, India, Brazil, Italy, France, and Russia are most affected by this pandemic. Currently SARS-CoV-2 is more infectious than previously known epidemic SARS virus (6). The current pandemic imposed by COVID-19 has caused more deaths in shorter period than epidemic SARS-CoV-1 lasted for 6 months causing <800 deaths (43). COVID-19 has a crude death rate of below 5% in global population whereas MERS and SARS had ~10 and 35% of mortality rate, respectively, (11). At present COVID-19 has higher mortality than influenza and seasonal influenza, however the accurate death rate would be available after 1 year. As the viral shredding takes place at 1–2 days post infection of SARS-CoV-2 hence the recovery rate is collectively high. The reproductive rate (R0) for COVID-19 was estimated by WHO was 2–2.5 (44).

## Virology of SARS-CoV-2 Virus

To date seven species of Coronaviruses has been identified and was found to be threatful to human respiratory system. Among them four species like hCoV-229E, OC43, NL63, and HKU1 are considered as less infectious whereas viruses like MERS, SARS, and SARS-CoV-2 are considered highly infectious and fatal (20). The SARS-CoV-2 is very closely similar to the SARS virus which was previously well known for causing epidemic of 2002–2003. The SARS-CoV-2 antigen or spike (S) protein is capable of binding to human angiotensin converting enzyme II (ACE2) receptor as it has structural similarity to SARS-CoV-1. This virus was found to be 79.6% similar to the SARS-CoV-1 (45). It was found that SARS-CoV-2 has 98.7% nucleotide homology to horseshoe bat coronavirus (46). The isolated strains of SARS-CoV-2 from infected patients had 99.98% similarity with each other, suggesting no antigenic drift noted during early phase of outbreak (18, 46). The SARS-CoV-2 viral genome is similar to coronavirus (CoV) strains indentified in pangoline and bats (47).

The SARS-CoV-2 virus failed to infect laboratory mice by gaining entry through the ACE2. However, this viral infection in mice could be achieved by cloning human-ACE2 (hACE2) in mice to obtain pathogenecity (48). ACE2 are widely expressed on T-lymphocyte cells of mammalian lungs (49, 50). Using Immunohistochemistry (IHC) a study demonstrated the expression pattern of ACE2 which is widely expressed in alveolar epithelial cells, bronchial epithelial cells, bronchial serous glandepithelial cells, monocytes/macrophages, gastric parietal cells, myocardial cells, distal convoluted renal cells, adrenal cortical cells, sweat gland of epithelial cells, and acidophilic cells of pituitary. The ACE2 were not expressed in bronchial mucous gland epithelial cells, follicular epithelial cells of thyroid and gastric chief cells (35). A study analyzed RBD (receptor binding domain) gene of 103 strains of SARS-CoV-2 found two types of molecular divergence in its genomes S (Serine) type and L (Leucine) type. The L-type strains was dominant and aggressive in nature compared to S-type was seen during the early phase of Wuhan outbreak (51).

The SARS-CoV-2 genome is differentiated into two basic types of non-structural proteins and structural proteins encoding genes. There are overall 16 non-structural proteins in orf1a/b encoded by this gene sequence necessary for suppressing host defense mechanism, replication, reverse transcription, helicase, host specific binding elements etc. The structural proteins are of four types spike (S), envelop (E), membrane (M), and nucleocapsid (N) proteins these proteins are synthesized by four known genes ORF2 (S), ORF4 (E), ORF5 (M), and ORF9 (F), respectively (20, 52). Additionally there are eight open reading frame genes named as ORF1ab, ORF1a, ORF3a, ORF6, ORF7a, ORF7b, ORF8, and ORF10 responsible for respective protein generation. The viral genome is responsible for producing sixteen (16) non-structural proteins these functions in inducing host mRNA cleavage (NSP1), binding to prohibitins (NSP2), proteinase activity (NSP3), membrane formation (NSP4), NSP polypeptide generation (NSP5), autophagosome generation (NSP6), dimerizing (NSP7), helicase (NSP9), stimulates NSP16 (NSP10), unknown (NSP11), RNA polymerase (NSP12), RNA caping (NSP14), endoribonuclease activity (NSP15), methylation, binding, and stimulation of NSP's (NSP16) (52). Among all these NSP1 is known for suppressing antiviral host response by inhibiting interferon (IFN) response genes and degrades host cellular mRNA (53).

## Prognosis

The levels of different cytokines in COVID-19 patients were closely monitored in hospitalized patients. The Interferon gamma-induced protein 10 (IP-10) levels could be potentially distinguished in the severe and non-severe hospitalized patients. Gradual rise or decrease in its level would suggest if patient would require prolong hospitalization or early discharge (23). The use of computerized tomography (CT) scan or magnetic resonance imaging (MRI) to confirm the bilateral opacities of patchy shadows present in patient's lungs caused due to viral infection followed by pneumonia could be confirmed, ARDS associated changes could be observed during the severe cases of COVID-19 (17, 54). Early identification of fungal or bacterial co-infection in COVID-19 cases would potentially increase the survival chances of the subjects.

## Clinical Symptoms of SARS-CoV-2

As per WHO, symptoms like fever, cough, sore throat, nasal congestion, malaise, headache, and muscle pain were observed in uncomplicated COVID-19 patients (3). The non-ICU patients presented high fever than the patients in ICU (1, 12). Possibly due to early and regulated secretion of pro-inflammatory cytokine like IL-1β in non-ICU patients reduced the probability of ICU admission. Symptoms like constipation and abdominal pain was rare in SARS-CoV-2 patients (12, 19). While symptoms like rhinorrhoea, diarrhea, and vomiting was commonly seen in hospitalized patients (12, 24, 25, 27). Liver dysfunction in 2–11% of patients was a frequent comorbidity, elevated hepatic enzymes was seen in 14–53% of infected cases (55).

## Immunopathology of SARS-CoV-2 Infection

Activation of lymphocytes and neutrophils during superinfection process can directly modify the diseased state. Hence monitoring these two cell types would reveal the disease progression in patients (56). However, the change in lymphocyte and neutrophils counts observed during acute respiratory infection severe and non-severe conditions are mainly triggered by the acute phase cytokine storm (57). In 2012 a cohort study conducted involving patients suffering from pneumonia infection or influenza. In the H1N1 infected patients it was observed that neutrophil-lymphocyte count ratio (NLCR) below 10 tend to get less hospitalized than the patients with NLCR above 10 (58). The lymphocyte and neutrophil count is the primary criteria under SIRS score to be monitored in hospitalized viral pneumonia patients (3).

In the current pandemic condition serological studies suggested that the patients requiring intensive care had significantly high level of neutrophil counts and low level of lymphocytes entirely opposite was seen in the discharged individuals (19, 22, 25, 27). In COVID-19 adult patients the lymphocyte counts remained average whereas in elderly patients found to be below the lower range of 1.2 × 10^9^/L (20). Fei Zhou et al. reported lymphocytes count was below 1.0 × 10^9^/L in 38 out of 137 patients (26). Studies have confirmed neutrophil count in ICU patients to be significantly higher than Non-ICU ones, while the lymphocyte count was not much different in these two groups of patients (1, 12). The lymphocyte count decreased in both critically ill survivor and non-survivor groups of COVID-19 (23, 27). The average lymphocyte count increased while the average neutrophil count in pediatric patients with non-severe SARS-CoV-2 pneumonia remained within normal range. The age of the patients ranged from 2 months to 15.6 years all the patients survived. The new born (>2 months old) and children <4 years among these nine children showed early recovery had highest level of lymphocyte and moderate level of neutrophils beside that all were positive for viral shredding post recovery from disease (21). The lymphocyte count and cytokine/chemokine levels in COVID-19 patients are illustrated along with the immunopathology in a chart (Table 1).

## Immunopathology of SARS-CoV-1 and Cap Infection

The progression of pneumonia in severe cases of SARS-CoV-1 or SARS-CoV-2 could be confirmed by evaluating the neutrophil count it could be high in serum but drastically increases in BALF samples (39). Isolation of BALF from patient lungs could be difficult mainly while an epidemic outbreak has already occurred as the patient intake in hospital could be unusually high. Due to the ability of CoV to infect and suppress the IFN induced defense mechanism in patients, this possibly resulted in depletion of T-cells leading to lymphocytopenia and T-cell exhaustion in adults (50, 53, 59).

It was reported that decrease in lymphocytes and increase in neutrophils could be visible in patients exposed to endotoxemia (60). In CAP and SARS-CoV-1 pneumonia patient's neutrophil count were higher than the average lymphocyte counts, similar to the SARS-CoV-2 pneumonia patients (1). Previously in SARS-CoV-1 infected patients leukopenia and lymphocytopenia were noted, SARS-CoV-1 affected individuals showed lower counts of overall T-cells, CD4+ T-cells, CD8+ T-cells, natural killer (NK) cells, and B-cells in serum samples (56). Also a study conducted in 2014 where they focused on both viral pneumonia and CAP patients showed minor difference between these two groups identified in the lymphocytes counts but not in the neutrophil counts. The neutrophil count remained high and equal in both viral and non-viral bacterial pneumonia groups (61). Later in a study's effort to distinguish SARS-CoV-1 pneumonia and CAP mediated changes found the neutrophil counts were substantially high in CAP cases than SARS-COV-1 pneumonia (severe and non-severe) cases but the lymphocyte count was reduced in SARS-CoV-1 but not in CAP group (37). Lymphocyte cells are responsible for producing significant amount of IFN-γ and tumor necrosis factor (TNF-α) in the SARS-CoV-1 infected patients (62). The lymphocyte count plays a decisive role during viral infection; patients with initial high level had lower risk of acquiring and dying due to pneumonia (24). The neutrophil and lymphocyte count shows similar trend in both SARS-CoV-1 and SARS-CoV-2 pneumonia patients (1).

## Immune Activation During Bacterial Pneumonia

Pneumonia can be caused by bacteria like *Streptococcus pneumoniae, Mycoplasma pneumonia, Klebsiella pneumoniae, Chlamydia pneumoniae, Staphylococcus aureus, Legionella pneumophila, Pseudomonas aeruginosa*, and *Haemophilus* influenzae type b (Hib) (58). *Streptococcus pneumoniae* is the most virulent pathogen capable of causing most fatal and common form of pneumococcal infection. Upon binding to the respiratory tract pneumococcal species further colonizes, multiplies, and spreads the infection in multiple regions of human body like ear (otitis), blood (bacteraemia), brain (meningitis) (63). In the existing pandemic viral pneumonia the most commonly identified bacterial species includes *Acinetobacter baumannii, Klebsiella pneumoniae*, and methicillin resistant *Staphylococcus aureus* (24).

The binding of the bacteria to the epithelial cells of bronchus or alveoli is the primary step toward infection. Upon the entry of bacteria in the bronchus through the nasopharyngeal space into the trachea moves further to alveoli, it infects the alveolar epithelial cells (AEC) this activates immune cells like macrophage and dendritic cells through cytokine release (64). Gram negative bacterium, lipopolysaccharides (LPS) induces IL-1β release from AEC it activates macrophage via NFkB mediated signaling pathway resulting in pro-IL-1β secretion (65). The proinflammatory cytokines activates the dendritic cells which in turn facilitates proliferation of the macrophage and neutrophils through the release of pro-inflammatory cytokines like tumor necrosis factor alpha (TNF-α), interleukin IL-1β, IL-6, IL-8, and IFN-γ (66).

The cytokines like IL-1RA, IL-6, IL-8, and IL-10 were found in initial phase of invasion, also known as acute phase cytokines (67). The initial titers of pro-inflammatory cytokine release leads to the activation of naive T-cells, it produces GM-CSF, G-CSF, IFN-γ, TNF-α, IL-1α/β, and IL-12 it enhances the ability to fight bacterial infection (38). The recruited T-cells play a significant role in bacterial clearance during the course of infection (68). T-cells are prominently known for IFN-γ production during respiratory viral infection but not during pneumonia (62). T-helper 17 cells play a vital role during bacterial infection by releasing IL-1β and IL-6; it activates and recruits neutrophils to the site of infection (69). Due to these above facts IFN-γ and IL-1β levels should be observed in patients with CAP. The levels of both pro-inflammatory and anti-inflammatory cytokines are important to be monitored in patients suffering from pneumonia.

Alveolar macrophage mediated inflammatory responses includes the cytokines release like IFN-α/β/γ, TNF-α, IL-1β, IL-6, and IL-8 these cytokines release causes fever, pain, headache and cough formation (70). The anti-inflammatory cytokines like IL-1 receptor antagonist (IL-1ra), transforming growth factor (TGF)-α/β and IL-10 plays a crucial role in suppressing the destructive function of inflammatory cytokines during critical condition (71, 72). The major role of pro-inflammatory cytokines IL-1β is to continuously recruit polymorphonuclear (PMN) neutrophils at the site of infection to effectively reduce the bacterial load this causes ALI and ARDS (73). IL-1β mediated chemokine (CXCL1/2) release is necessary for suppressing infection via neutrophil activation, similarly IL-8 mediated CXCL2 release simultaneously triggers neutrophil activation and proliferation (74, 75). Thus, increased IL-1β level has negative impact on patient's survival during severe CAP, IL-1ra, and IL-8 release downregulates the negative impact of IL-1β (69, 76).

The disrupted epithelial cells and macrophages signals the proliferation of neutrophils causing the accumulation of cellular debris along with blood, neutrophils and macrophage forms fluid in the alveoli giving arise to pulmonary edema (70, 77). Failure of neutrophils to degrade the bacteria within it via phagocytosis mediated degradation could increase the bacterial load causing neutropenia increasing the criticality in patients often observed during multi drug resistant (MDR) pneumonia (78). The up-regulation of granulysin and perforin mediated bacterial cell lysis beside that the neutrophil extracellular traps (NET), ROS/NOS, anti-microbial peptides and protease enzyme produced by the alveolar macrophages also by PMN neutrophils all together terminates the bacteria (70, 75).

## Immune Activation During Fungal Infection

Presently fungal pneumonia is seen in patient confirmed to be *Candida glabrata, Candida albicans*, and *Aspergillus flavus* (24). During the course of fungal pneumonia dendritic cells and macrophages activates the NK cells through release of IL-12 (79). Sequentially NK cells IFN-γ release leads to maturation of Th1 cells. On the other side dendritic cells causes proliferation of Th17 cells by secreting TGF-β, IL-6, IL-1β, IL-21, and IL-23. Both the activated T-helper cells mediated neutrophil maturation and proliferation at the fungal infection site. The cytokines level of IFN-γ, IL-12, IL-17A/F, IL-23, and IL-22 has protective role in neutralizing the fungal invasion (80). Activation of neutrophil leads to similar complications like bacterial pneumonia, however early and balanced proliferation of neutrophils is important for patient's survival. Fungal pneumonia has differential observations from bacterial pneumonia in ground glass opacities (GGO) obtained from the CT-scan report (81).

## Cytokines Imbalance Leads to Viral Pneumonia

The viral pneumonia is a co-infection caused initially by any virus like influenza A/B viruses, respiratory syncytial virus, enterovirus, adenovirus, rhinovirus, metapneumovirus, parainfluenza virus, bocavirus, MERS and SARS coronaviruses, in association with secondary bacterial or fungal infection results in fatality, it is also known as superinfection. This type of co-infection was commonly seen in the previous influenza virus pandemic of 2009 (10, 82). The viral entry causes disruption of alveolar epithelial cells (AEC), it cascades the signal for production of cytokines like granulocyte/macrophage colony-stimulating factor GM-CSF it signals maturation and recruitment of monocyte and macrophage cells (83). The production of TNF-α by macrophage cells present at very low level during acute infection phase within the alveoli, signals AEC to produce GM-CSF (84). Later the activated and matured macrophages play a vital role in proliferation of dendritic cells and recruitment of macrophages which all together leads to chemotactic recruitment of neutrophils (85). GM-CSF is produced by AEC has a prominent role in recruitment and activation of dendritic cells important for adaptive immune response, it promotes the raising level of alveolar macrophages mandatory for innate immune response to tackle bacterial invasion (68). All the viruses mainly targets lymphocytes for its growth and replication.

Acute respiratory infection caused by viruses sub-sequentially engages the immune cells at the site of infection and changes the composition of the lungs and gastro intestinal tract known as dysbiosis. While suppressing the viral load the opportunistic bacteria or fungi residing in the nasopharyngeal space invades easily the alveoli of the lungs (86). The predepository factors attributed by influenza virus or coronavirus infection is mainly mitigated by the release of IFN-γ, it downregulates IL-1β level necessary to fight against bacterial or fungal infection demonstrated in mice (87). In another study shown in mice model the derogatory role of IFN-γ initiates after it binds with macrophage cells and impairs neutrophil recruitment during bacterial co-infection (88). Similarly using a murine model it was revealed that IL-1β production is inhibited by IFN-γ during influenza infection (89). It is further reviewed here if the mechanism of dysbiosis exists in SARS-CoV-2 mediated cytokine storm leading to cytokine imbalance and pneumonia progression. The IFN-γ level shoots high during pneumococcal pneumonia but not in the case of staphylococcal pneumonia, so alternatively IP-10 level would be suitable to observe IFN-γ mediated changes in clinical studies (90, 91). In severe/fatal scenario of SARS-CoV-2 infection causes imbalance of IFN-γ and IL-1β cytokine levels and related molecular pathways resulting in dysbiosis is yet to be studied.

## Cytokines Levels in Pneumonia Patients

During the severe pneumonia it was observed that level of IL-1β was at higher concentration during fatal condition than non-fatal patients. The proinflammatory cytokine level tends to double in the BAL fluid than in the blood of viral pneumonia challenged individuals (60). The patient's BAL fluid had elevated IL-1β than in serum due to presence of bacterial infection, IFN-γ level was not confirmed (28). The IFN-γ level was found to be similar compared to healthy pediatric patients infected with *Mycoplasma pneumoniae* pneumonia found in a meta-analysis (92). IFN-γ levels were found to be normal in a study involving children suffering from severe pneumonia (38). In a study focusing on patients infected by *Legionella* pneumonia found low level of IL-1β except the fatal case, the level of IFN-γ elevated during acute phase but it diminished with time (30). The CD8+ T-Cells are responsible for producing and maintaining high levels of IFN-γ required for clearing the viral load at the same time IFN-γ plays a major role in recruitment of T-cells, but its level remains insignificant during non-severe CAP (93).

In severe CAP (where three out of 10 patients suffered from Haemophilus influenzae and H1N1) both the BAL fluid and serum cytokine level showed high IFN-γ and low IL-1β levels in CAP patients. Severe CAP group compared to non-severe CAP shows high level of IFN-γ but not IL-1β suggests possible role of dysbiosis causing this imbalance during co-infection. Cytokines like IL-8 and IL-1β are responsible for the activation of neutrophil, during severe pneumonia infection these were found to be elevated in BALF of fatal cases (29). The IL-1β levels were elevated in severe CAP patient's BAL fluid samples, possibly because this study enrolled both pneumococcal pneumonia and viral pneumonia patients. Thereby, it is possible that due to lymphocytopenia or leukopenia caused by any of the other comorbid conditions may raise the chances of dysbiosis resulting in pneumonia.

## Cytokines Imbalance in SARS-CoV-1 Pneumonia Patients

The cytokines level often changes depending on the type of bacterial and fungal species. Difference in cytokine level could be observed when groups are sorted as per secondary infections (90, 92). The IL-1β level was found to be ~25–49% higher in dead patient's AEC upon SARS-CoV-1 infection, whereas TNF-α level was reported to be around 50–75% higher in same fatal cases, IFN-γ level was not measured in fatal cases by IHC (35). In a study conducted in Hong Kong included 20 patients infected with SARS-CoV-1, under non-severe scenario the serum IL-1β level was significantly higher (above 1.3 ng/L considered significant) for 13 days post infection until recovery, whereas it was not observed in IFN-γ level (above 15.6 ng/L considered significant) in same patients while monitored for 18 days, it is possibly because both severe and non-severe patients were included in the same group (33). The IL-1 gene expression pattern measured in SARS-CoV-1 severe and non-severe patients did not vary with results for IL-1β measured by enzyme immuno assay (EIA) (94, 95).

The IFN- γ level was not found to be high in SARS-CoV-1 group (one patient had ARDS), but substantially elevated in CAP group, however the IP-10 level was higher in SARS-CoV-1 infected individuals than CAP group (37). The level of IFN-γ in end stage of severely infected SARS-CoV-1 patients was not substantially different from normal patients, but IP-10 level increased in fatal cases than in normal or convalescent groups. There is a clear indication from above clinical data that presence of high viral load caused prolonged activity of IFN-γ and viral replication resulted in lymphocytopenia. IL-1β level did not changed in hospitalized SARS-CoV-1 infected patients but was increased in fatal cases detected by mRNA expression (31). IL-1β leads to neutrophil activation necessary during acute phase of pneumonia, slower secretion of IL-1β increases bacterial growth resulting in faster disease progression. Viral load never declines in severely infected viral pneumonia fatal cases. The failure in up-regulation of signature level of IL-1β is necessary for suppressing the bacterial load causes dysbiosis it increases complications or results death (86).

The serum level of IL-1α and IFN-γ were found at non-significant level among severe and non-severe SARS-CoV-1 patient groups. Anti-inflammatory cytokines like TGF-β and IL-10 level increased in convalescent group (40). IL-1β level in non-severe SARS-CoV-1 infected children were substantially high before and after the treatment with anti-inflammatory drugs. The IL-1β level was reported to be induced by the activation of caspase-1 dependent pathway triggered by viral entry in macrophage cells (32). The lymphocytes count trends to decline during SARS-CoV-1 and SARS-CoV-2 viral pneumonia suggesting IFN-γ release from alternative cellular sources. IFN-γ is produced by NK cells, T-cells and antigen presenting Cells (APC) during viral infection, suggesting NK cells and APC are alternative source of IFN-γ during coronavirus infection (88).

Kao-Jean et al. reported that IFN-γ level was not substantially different in the SARS-CoV-1 fatal and non-fatal cases, but the level of IFN-γ was several fold higher in acute stage of viral infection in patients than normal individuals (39). IL-1β increased with severity of SARS-CoV-1 in critical patients while its levels remained normal in non-severe patients (34, 36). The mechanism of dysbiosis could be seen in these clinical studies with SARS-CoV-1 pneumonia critical patients. The imbalance of IL-1β and INF-γ/IP-10 in CAP (severe), SARS-CoV-1 (severe and non-severe) and SARS-CoV-2 (severe and non-severe) are shown in Table 2.

## Cytokines Imbalance in SARS-CoV-2 Pneumonia Patients

The cytokine storm taking place in SARS-CoV-2 infected patients suggested that pro-inflammatory cytokine IL-6 and anti-inflammatory IL-10 levels to be substantially elevated in both mild and severe cases (24). Cholin Huang reported high level of IL-2, IL-7, IL-10, G-CSF, IP-10, TNF-α, MCP-1, and MCP-1A increased in ICU patients with SARS-CoV-2 pneumonia (1). Previously in middle east respiratory syndrome coronavirus (MERS-CoV) severely infected patients the levels of IFN-γ, TNF-α, IL-15, and IL-17 were reported to be high (96).

The immediate recruitment of neutrophil is required during pneumonia, delayed activation are due to ongoing fight against viral invasion delays the GM-CSF production (83). The TNF-α and IL-1 mediated GM-CSF production induces controlled neutrophil recruitment and vice versa (97, 98). Additionally GM-CSF enhances survival, proliferation and phagocytosis of immune cells, GM-CSF plays an important role during the life threatening cases of sepsis (68). GM-CSF was not significantly high in ICU admitted patients of SARS-CoV-2 compared to the non-ICU patients; this suggests the importance of GM-CSF during the early phase of infection in severely affected patients (1). Beside that the T-cell decreases the viral load during CoV invasion, but the T-cells are the prime target of this virus (99).

Elevated level of IFN-γ was a usual marker for SARS-CoV-2 infected severe as well as in non-severe groups, but no major difference between severe or non-severe groups. The level of IL-1β was significantly high in ICU patients compared with healthy individuals however the IL-β level was below 2 pg/ml. Increased IP-10 level was related to high IFN-γ activity which was high in ICU patients. IL-1β level remained in normal range for healthy individuals but increased in both ICU and non-ICU (low statistical significance) held patients the patient's serum which were collected soon after admission and was not regularly monitored (1). A study sorting SARS-CoV-2 pneumonia groups as per the requirement of MV considered critical, while non-invasive MV considered as severely ill and least MV requiring patients grouped as moderate. Upon prolong monitoring of IL-1β for 24 days post infection in these 3 focus groups where statistical significance in critical and severe patients was seen on 15th day. The IL-1β level decreased in critical group suggesting disease progression while IP-10 level remained high even after prolonged treatment with immunosuppressant observed 15 days after onset. The increasing level of IP-10 and MIG seen in these groups modulates the disease and signals toward severe condition of lungs, while decreased level was positively correlated with healing when IP-10 was below 1,000 pg/ml in serum. The IP-10 and MIG genes are induced by IFN-γ activity during viral pneumonia. It was reported that reduced IL-1β level in critically ill ICU patients compared to less critical ones, in that study G-CSF, M-CSF, CTACK, IL-18, IL-13, MIP-1α, MIP-1β, MCP-3, MIG, HGF, IL-1ra, IL-1β, and IP-10 were monitored regularly in patients (23). Similarly observed in influenza-pneumonia and CAP there was clear indication that IFN-γ downregulates the IL-1β level similar trend is observed in COVID-19 pandemic but further study is required at molecular level in future to draw this conclusion. This calls for further study to reveal the underlying intercellular mechanism causing down regulation of IL-1β by IFN-γ in critically ill patients.

During severe instance of sepsis the IFN-γ level rises and it has detrimental effect on individual life expectancy. In the same study it was reported that IFN-γ single nucleotide polymorphism gives rise to increased susceptibility to pneumonia in Chinese population (100). Similarly IL-1β gene polymorphism was observed in Iraqi children found increased susceptibility to pneumonia infection. It was observed in the study that IL-1β level was not substantially increased in CAP patients compared to healthy individuals (101). Further it was noted that IL-10 encoding SNP genes were found responsible for exacerbating systemic inflammatory response syndrome (SIRS) score during CAP infection but not the TNF-α and IL-6 cytokines (102). Any of these SNP gene mutations in COVID-19 patients play an important role in progression of pneumonia. Immunosuppressive corticosteroids could be effectively used to reduce the burden of SIRS. The levels of the cytokines, interferons and chemokines were GM-CSF, G-CSF, TNF-α, IFN-γ, IL-1β, IL-2, IL-4, IL-6, IL-7, IL-8, IL-10, IL-12, IL-17A/F, IL-22, and IP-10 were monitored in SARS-CoV-2 pneumonia patients (1). Excess production of IL-1β has negative effect on patient's survival and disease recovery but early induction of this pro-inflammatory cytokine possibly leads to early or quick recovery.

## Cytokine Levels in Severe SARS-CoV-2 Children and Aged (Females and Males)

In most recent study supporting the concept of dysbiosis could be observed where pediatric patients suffered from SARS-CoV-2 pneumonia on an average the level of IFN-γ increased while IL-1β level remained normal during the course of treatment in hospital with antivirals and interferon-alpha (IFN-α). The SARS-CoV-2 infected children <4 years demonstrated early recovery with increased viral shredding for prolonged time where lymphocyte and IFN-γ level elevated although neutrophil and IL-1β level remained within normal range (21). This evidence collectively suggested children <4 years accommodate virus possibly due to higher lymphocyte count promoting constant viral replication for several days post recovery and required prolong monitoring of viral titer. The lymphocytes level in children within 1 month to 3 months remains high due to enhanced activity of thymus but it trends to decline with old age, it could be a possible reason for reduced deaths of children by COVID-19 pneumonia (103). Similarly IL-17F and IL-22 were found significantly higher in children, these two cytokines protects during fungal invasion however fungal pneumonia was not reported in the pediatric patients (21). The Th2 cells are well known for secreting IL-17F and IL-22 during viral infection (80).

In mice model it was demonstrated upon *Acinetobacter baumannii* infection of lungs, resulted in death of aged mice but not young mice, even vaccination against this species did not protected the older mice (104). This specific superinfection was common in COVID-19 patients of Wuhan; its presence exacerbates to irreversible condition and leads to confirmed death. In the current pandemic, women are less fatally affected by the virus than men. Possibly due to the fact that in females of age group above 50 years has higher lymphocyte counts compared to males of same age group is responsible for protection against infection. In this study to further demonstrate gender difference of withstanding infection found higher leukocyte count in Chinese males than its female counterpart (105). Sexual dimorphism in immunity was seen in female mice with enhanced disease fighting abilities against Staphylococcus induced peritonitis than the male mice (106).

## Treatment Strategy

The treatment strategy involves the use of antiviral medicines to tackle the viral loads in coronavirus infected patients. However, certain antivirals are ineffective in treating the COVID-19 patients, drugs like ganciclovir, acyclovir and ribavirin may not prove effective in patients. Drugs like neuraminidase inhibitors and protease inhibitor lopinavir/ritonavir along with IFN-α in combination were reported to be effective in SARS-CoV-2 challenged patients (19). The antiviral oseltamivir effective against influenza was found effective in reducing SARS-CoV-2 viral load upto a greater extent (1). Antibiotics against pneumococcal infection could induce effective suppression of bacterial growth in the patients with pneumonia. Antibiotics like amoxicillin, azithromycin, and fluoroquinolones were in use for reducing bacterial burden in patients (19, 107). Antiviral agents like umifenovir, remdesivir, and chloroquine were found to be effective against SARS-CoV-2 in patients (108). Drugs like cephalosporins, quinolones, carbapenems, and tigecycline were successfully used against methicillin resistant *Staphylococcus aureus* along with linezolid and other antifungal drugs showed inhibitory activity against pneumonia in severely infected patients (24). Corticosteroid like methylprednisolone, dexamethasone, and methylprednisolone sodium succinate along with intravenous immunoglobulin therapy were reported to be in use for patients suffering from MDR pneumonia (19). Heparin was recommended after successfully demonstrated clinically to reduce coagulopathy in patients with sepsis (108). In a non-randomized study the serum transfusion from COVID-19 recovered or convalescent patient to SARS-COV-2 infected critically ill patients recovered within 11–13 days post transfusion (109). Cytokine absorption devices are currently in use for managing critical conditions; it eliminates the excess amount of circulating cytokines in the blood of SARS-CoV-2 pneumonia patients (110).

**Figure 2:**
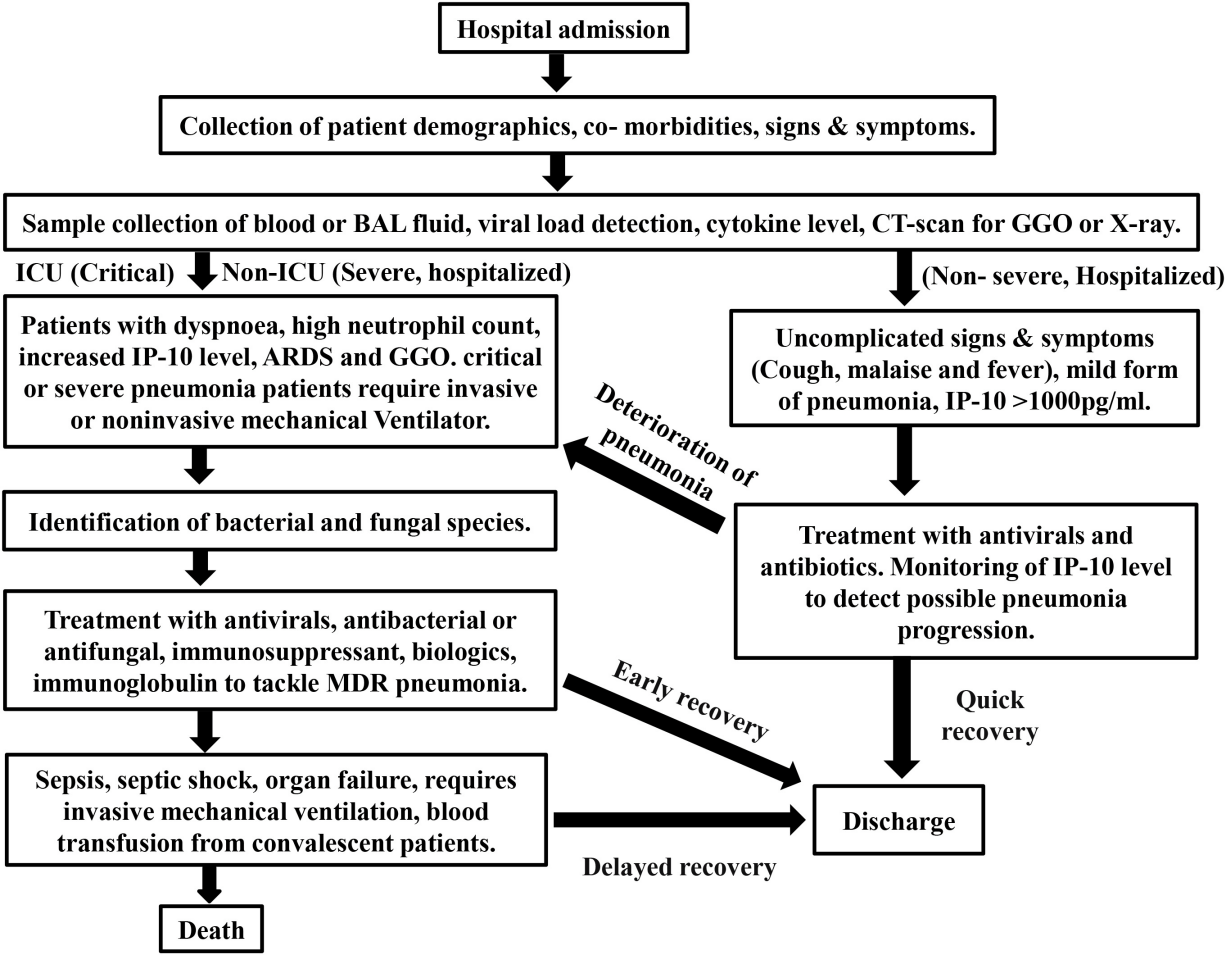
Flow chart for prognosis and treatment strategy for COVID-19.

Interferon-γ level has negative effect on patient's susceptibility to withstand severe condition like bacterial sepsis and not recommended by WHO for the existing COVID-19 treatment (111). Cellular therapy involving the cytokines like GM-CSF was clinically proven and suggested (112). IFN-α is more suitable for SARS-CoV-1, SARS-CoV-2, and MERS virally challenged individuals as demonstrated in mice (113, 114). Early inhalation of GM-CSF and treatment using IL-6 inhibitor (tocilzumab) for patients suffering from viral pneumonia has repressive role in sepsis progression (112, 115). The treatment strategy, essential cytokine markers, disease progression and prognosis are described in brief with a flow chart (Figure 2).

The IL-1 inhibitor (anakinra) showed patients treated with it recovered from the COVID-19-pneumonia. Two different clinical studies demonstrated anti-IL-1 receptor has positive impact on patient's survival. In a non-randomized study set up patients treated with anti-IL-1 receptor antagonist saved significant number of lives. All the eight patients were comorbid with hemophagocytic lymphohistiocytosis (HLH) a rare immune disorder responsible for high morbidity. Increased number of lymphocyte and macrophage cells is commonly seen in patients with HLH. By treating with anakinra 300 mg/day for seven days, four patients required mechanical ventilator, three were treated with corticosteroids and one attended quick recovery among them three patients died (116). This study suggested that hyperactivated immune system may delay the recovery process in patients. A clinical study could be conducted further to confirm the efficacy of IL-1 inhibitor beside corticosteroid treatment group in severely ill patients. Similarly, a cohort study involving COVID-19 patients comparing with a historical placebo group with the anti-IL-1 treated group found reduced number of deaths in treatment group. In this study none of the patients suffered from cancer but suffered from other comorbidities and were treated with 200 mg/day for 3 days than 100 mg/day for seven days (117). Both studies involving SARS-CoV-2 pneumonia patients suggested further studies should be conducted to conclude the efficacy of anti-IL-1 antibodies. In future randomized clinical study including patients suffering from multidrug resistant SARS-CoV-2-pneumonia should be enrolled and treated with anti-IL-1 to identify its efficacy.

## Limitations

The studies discussed in this review mostly focused on studies of Chinese population and published during the early phase of pandemic. The initial review of literature search was limited to pubmed, other repository or databases were not searched. The cytokine storm seen during epidemic SARS-CoV-1 and initial wave of SARS-CoV-2 was mainly highlighted to review the cause of dysbiosis. The cytokine levels could be diverse in populations of different global regions affected by COVID-19 due to varying secondary infections is not reviewed here. The immunopathological trends should remain the same and may not vary significantly. The side effects attributed by treatment regimen in COVID-19 patients are not discussed here. Few of the COVID-19 literatures discussed here measured the cytokine level upon admission to the hospital (1). Few authors reported patients were monitored for cytokines levels throughout the hospitalization phase and was treated with antivirals, immunosuppressant and interferon-alpha (IFN-α) (23). Accurate mortality rate of COVID-19 and basic reproduction number of SARS-CoV-2 are yet to be estimated. The level of cytokine in COVID-19 non-hospitalized patients is not discussed here.

## Conclusion

Signature cytokine levels of IL-1β is an important factor activated during acute phase of viral pneumonia, as its level may not be significant obtained from large number of patients infected by varying bacterial or fungal species (1, 92). It is presently seen in fatal cases that homeostasis of immune system is not maintained resulting in imbalance of immunological cells and cytokine levels mainly IL-1β and IFN-γ during dual mode of infection such as viral pneumonia increases the complications in COVID-19 patients (87, 118). Low level of lymphocytes mainly Th2 cells during the acute phase of viral infection causes failure in recruitment of anti-bacterial neutrophils required to tackle the secondary infection (69). In the severe cases of viral pneumonia patients died due to bacterial burden but not due to primary viral infection (12). Observed in MERS, SARS-CoV-1, and COVID-19 patients it is understood that initially delayed but gradual increase of IL-1β complicates patient's condition (23). Increase in cytokines levels of IL-1β, IFN-γ, IL-6, IL-8, and IL-10 are the main cause and sign of disease progression (11). Pre-acquired gene polymorphism of IL-1β or IFN-γ genes in COVID-19 patients and superinfection by *Acinetobacter baumannii* in elderly COVID-19 patients may raise the chances of death irrespective of known co-morbidities.

Hereby it is suggested that similar to MuLBSTA score and SIRS score, the chemokine (IP-10) fold change, along with lymphocyte and neutrophil counts should be closely monitored for early detection and progression of SARS-CoV-2 pneumonia (3, 119). The initial level of white blood cells (WBC) types, cytokines profiles along with the GGO lesions detected in CT-scan report, beside the rectal swab test done to detect viral shredding are useful for prolong monitoring in patients (25, 120). The isolation and identification of specific fungal or bacterial species would help in precise treatment with antibiotics (54). During viral pneumonia pandemic it was recommended to collect the BAL fluid for immunological studies rather than collection of blood samples (60). As observed in MERS, SARS-CoV-1, and COVID-19 patients it is understood that initially delayed but gradual increase in cytokines like IL-1β, IFN-γ, IL-6, IL-8, and IL-10 is the main cause of rapid sepsis progression (11). Immuno-compromised patients suffering from viral pneumonia are subjected to prolonged mechanical ventilation either invasive or non-invasive it may prove to be fatal or worsen the disease condition prolonging the recovery time (77). Targeting the GM-CSF-IL-1β axis in humans could be an effective method to attain therapeutic benefit during early phase of viral pneumonia which is explored clinically (NCT04569877).

## Author Contributions

The author confirms being the sole contributor of this work and has approved it for publication.

## Conflict of Interest

The author declares that the research was conducted in the absence of any commercial or financial relationships that could be construed as a potential conflict of interest.
